# Analysis of TCR Repertoire and PD-1 Expression in Decidual and Peripheral CD8^+^ T Cells Reveals Distinct Immune Mechanisms in Miscarriage and Preeclampsia

**DOI:** 10.3389/fimmu.2020.01082

**Published:** 2020-06-03

**Authors:** Keiko Morita, Sayaka Tsuda, Eiji Kobayashi, Hiroshi Hamana, Kei Tsuda, Tomoko Shima, Akitoshi Nakashima, Akemi Ushijima, Hiroyuki Kishi, Shigeru Saito

**Affiliations:** ^1^Department of Obstetrics and Gynecology, University of Toyama, Toyama, Japan; ^2^Department of Immunology, Faculty of Medicine, Academic Assembly, University of Toyama, Toyama, Japan; ^3^University of Toyama, Toyama, Japan

**Keywords:** effector memory CD8^+^ T cell, human pregnancy, miscarriage, preeclampsia, T cell repertoire

## Abstract

CD8^+^ T cells, the most abundant T cell subset in the decidua, play a critical role in the maintenance of pregnancy. The majority of decidual CD8^+^ T cells have an effector memory phenotype, while those in the peripheral blood display a naive phenotype. An increased amount of highly differentiated CD8^+^ T cells in the decidua indicates local antigen stimulation and expansion, albeit these CD8^+^ T cells are suppressed. In decidual CD8^+^ T cells, co-inhibitory molecules such as PD-1, TIM-3, LAG-3, and CTLA-4 are upregulated, reflecting the suppression of cytotoxicity. Previous studies established the importance of the PD-1/PD-L1 interaction for feto-maternal tolerance. CD8^+^ T cells could directly recognize fetal-specific antigens, such as HLA-C, expressed by trophoblasts. However, although fetal-specific CD8^+^ T cells have been reported, their TCR repertoires have not been identified. In this study, we analyzed the TCR repertoires of effector memory CD8^+^ T cells (CD8^+^ EM cells) and naive CD8^+^ T cells (CD8^+^ N cells) in the decidua and peripheral blood of women with normal or complicated pregnancy and examined PD-1 expression at a single-cell level to verify whether antigen-specific CD8^+^ T cells accumulate in the decidua and to identify immunological differences related to the suppression of antigen-specific CD8^+^ T cells between normal pregnancy, miscarriage, and preeclampsia. We observed that some TCRβ repertoires, which might recognize fetal or placental antigens, were clonally expanded. The population size of clonally expanded CD8^+^ EM cells was higher in the decidua than in the peripheral blood. CD8^+^ EM cells began to express PD-1 during the course of normal pregnancy. We found that the total proportion of decidual CD8^+^ EM cells not expressing PD-1 was increased both in miscarriage and in preeclampsia cases, although a different mechanism was responsible for this increase. The amount of cytotoxic CD8^+^ EM cells increased in cases of miscarriage, whereas the expression of PD-1 in clonally expanded CD8^+^ EM cells was downregulated in preeclampsia cases. These results demonstrated that decidual CD8^+^ EM cells were able to recognize fetal-specific antigens at the feto-maternal interface and could easily induce fetal rejection.

## Introduction

Immune tolerance to the “semi-allogeneic” fetus is particularly important for successful pregnancy. Decidual CD4^+^ T cells, CD8^+^ T cells, and NK cells are activated during pregnancy (1); in particular, decidual CD4^+^ T cells are activated when an HLA-C mismatch is present between the mother and the fetus (2). Maternal regulatory T cells (Treg cells) play an important role in the maintenance of pregnancy by preventing rejection (3, 4). During pregnancy, CD8^+^ T cells become predominant among decidual immune cells and play a major role in feto-maternal tolerance. The main population among decidual CD8^+^ T cells (dCD8^+^ T cells) is represented by effector memory CD8^+^ T cells (CD8^+^ EM cells) that are thought to potentially induce fetal rejection, while the predominant population among peripheral CD8^+^ T cells (pCD8^+^ T cells) are naive CD8^+^ T cells (CD8^+^ N cells) (5). Previous studies revealed functional differences between decidual and peripheral CD8^+^ T cells. Decidual CD8^+^ EM cells (dCD8^+^ EM cells) exhibit higher production of interferon-γ (IFN-γ) and interleukin-4 (IL-4), as well as reduced perforin and granzyme B expression, compared to peripheral CD8^+^ EM cells (pCD8^+^ EM cells) (5, 6). dCD8^+^ EM cells express higher levels of inhibitory checkpoint molecules such as programmed cell death-1 (PD-1), T cell immunoglobulin mucin-3 (TIM-3), lymphocyte-activation gene-3 (LAG-3), and cytotoxic T lymphocyte associated protein-4 (CTLA-4) compared to pCD8^+^ EM cells (6–8). A high PD-1 expression was reported in decidual immune cells such as CD8^+^ T cells, regulatory T cells (Treg cells), and NKT-like cells (7, 9, 10), and programmed cell death ligand-1 (PD-L1) was found to be highly expressed in extravillous trophoblasts (EVT), syncytiotrophoblasts (ST), and other immune cells at the feto-maternal interface (11–15). The blockade of the PD-1/PD-L1 pathway results in increased fetal resorption in mice (16), suggesting that this axis is necessary for immune tolerance in the decidua. Therefore, the cytotoxicity of CD8^+^ T cells in the decidua is regulated so as to promote immune tolerance against fetal antigens during pregnancy, albeit these cells maintain a cytotoxic potential against virus-infected cells (17).

Immunological differences have been reported between normal pregnancy, miscarriage, and preeclampsia. Ramhorst et al. demonstrated that in non-pregnant women undergoing recurrent pregnancy loss, the proportion of effector memory T cells in the peripheral blood is higher than in fertile non-pregnant women (18). Several studies reported that miscarriage and preeclampsia are associated with a decreased number of Treg cells (4, 19–23). Interestingly, clonally expanded decidual Treg cells are decreased in preeclampsia but not in miscarriage (24). In light of this increasing evidence, successful pregnancy seems to require an appropriate functional change in cytotoxic CD8^+^ T cells as well as a correct balance between cytotoxic CD8^+^ T cells and Treg cells.

Paternal antigen-specific tolerance is necessary for the maintenance of allogeneic pregnancy (4). Previous studies have identified fetal antigen-specific CD8^+^ T cells and Treg cells in mice (25, 26). However, the detection of fetal antigen-specific CD8^+^ T cells and Treg cells is technically difficult in humans, because of the high diversity of CDR3 amino acid sequences in TCRβ, with a lower boundary of 2 × 10^7^ in young humans (27). We have previously reported the existence of clonally expanded Treg cells by performing single-cell DNA sequencing of T cell receptor β (TCRβ) (24). The population size of clonally expanded Treg cells that are able to recognize fetal antigens at the feto-maternal interface is increased in the decidua, but not in the peripheral blood (24). In serial pregnancies, Treg cells expressing the same TCR clonotypes across different pregnancies were observed in the decidua, suggesting that these cells might recognize fetal antigens (24). The clonal population of decidual effector Treg cells is less abundant in preeclampsia than in normal late pregnancy, suggesting that paternal antigen-specific tolerance mediated by Treg cells might be disturbed in this condition (24). As CD8^+^ T cells can recognize fetal antigens at the feto-maternal interface, we hypothesized that antigens recognizing CD8^+^ T cells would be clonally expanded in the decidua, but that their function would be suppressed during human pregnancy. In addition, we postulated that the maldistribution or functional alteration of antigen-specific CD8^+^ T cells could underlie pregnancy complications.

In this study, we analyzed the TCRβ repertoire of decidual and peripheral CD8^+^ EM cells and CD8^+^ N cells in women undergoing normal pregnancy and in cases of miscarriage or preeclampsia. We further examined the expression of PD-1 in these cells, to clarify whether antigen-specific CD8^+^ T cells accumulated in the decidua, and to identify the mechanisms underlying their suppression during normal pregnancy, miscarriage, and preeclampsia. If decidual CD8^+^ T cells recognize fetal antigens, CD8^+^ T cells with the same TCRβ repertoire should be clonally expanded, and express elevated levels of PD-1 during normal pregnancy. Therefore, a difference in the proportion of antigen-recognizing CD8^+^ cells or in the expression of PD-1 should be observable between normal pregnancy, miscarriage, and preeclampsia.

## Materials and Methods

### Blood and Tissue Samples

Paired samples of peripheral blood mononuclear cells (PBMC) and decidual tissues were collected from 10 cases of artificial abortion in the 1st trimester (1st trimester normal pregnancy), 6 cases of miscarriage with normal fetal chromosomes in the 1st trimester (1st trimester miscarriage), 9 cases of uncomplicated pregnancy with delivery in the 3rd trimester (3rd trimester normal pregnancy), and 9 preeclampsia cases with delivery in the 3rd trimester (3rd trimester preeclampsia). As a control group, 6 samples of peripheral blood from age-matched healthy donors who had never been pregnant were collected. Written informed consent was obtained from all women in accordance with a protocol approved by the Ethical Review Board of the University of Toyama (Rin-28- 144). In the artificial abortion cases, the fetal heartbeat was confirmed before dilation and evacuation. For miscarriage cases, the diagnosis was formulated when the fetal heartbeat was lost or when the fetal heartbeat had not been detected inside the gestational sac for at least 2 weeks. In miscarriage cases, isolated chorionic villi were examined for fetal chromosomal karyotype by G-band staining, and only cases with normal fetal chromosomes were enrolled. The diagnosis of preeclampsia was based on the guidelines of the International Society for the Study of Hypertension in Pregnancy (28). Both the peripheral blood (10 mL) and the decidual tissues were obtained at dilation and evacuation, or after vaginal delivery or elective cesarean section. First-trimester decidual samples were derived from uterine content. Third-trimester decidual tissues were macroscopically dissected from the maternal surface of the placenta. The clinical and demographic characteristics of the enrolled patients are summarized in Table 1.

**Table 1:** Demographic and clinical characteristics.

	**Control**	**1st trimester**		**3rd trimester**	
	**No pregnancy history**	**Normal pregnancy**	**Miscarriage with normal fetal chromosomes**	**Normal pregnancy**	**Preeclampsia**
	**(*n* = 6)**	**(*n* = 10)**	**(*n* = 6)**	**(*n* = 9)**	**(*n* = 9)**
Maternal age (years)^a^	30 (25–35)	26 (22–39)	34 (26–41)	32 (22–36)	36.5 (28–41)
Body Mass Index (kg/m^2^)^a^	NA	18.6 (16.7–21.2)	21.4 (16.4–27.6)	18.4 (17.7–24.9)	23.7 (17.6–26.0)
Gravidity^a^	0 (0–0)	4 (1–7)	4 (3–7)	2 (1–3)	2 (1–5)
Parity^a^		2 (0–4)	0.5 (0–2)	1 (0–2)	0 (0–3)
Live birth^a^		2 (0–4)	0.5 (0–3)	1 (0–2)	0 (0–3)
Miscarriage^a^		0 (0–3)	2 (0–4)	0 (0–1)	0 (0–4)
Still Birth *n* (%)		0 (0.0)	0 (0.0)	2 (22.2)	0 (0.0)
Nullipara *n* (%)		3 (30.0)	3 (50.0)	4 (44.4)	6 (66.7)
Gestational week (weeks)^a^		8 (6–9)	8 (6–8)	38 (37–40)	35.5 (32–39)
Cesarean section (patient number) *n* (%)				5 (55.6)	5 (55.6)

a *Data are presented as median (range). NA: not available. Steel–Dwass test and Fisher's exact test were used for continuous and categorical variables, respectively. No statistically significant differences were observed between the groups*.

### Mononuclear Cell Isolation

Peripheral blood samples were layered on Ficoll Hypaque gradients (LymphoprepTM; Alere Technologies, Norway) for density gradient centrifugation (453 × g for 30 min). Mononuclear cells were isolated and washed twice with phosphate-buffered saline (PBS). Decidual tissues were rinsed thoroughly with PBS and minced into 1–2 mm pieces by a pair of scalpel blades in Dulbecco's Modified Eagle Medium. Then, the suspensions were filtered through a 32 μm nylon mesh as reported elsewhere (24). All samples were cryopreserved until single-cell analysis.

### Single-Cell Sorting

The following monoclonal antibodies were used for cell staining: anti-CD3 (FITC; BD Biosciences, San Jose, CA, USA), anti-CD8 (APC; eBioscience, San Diego, CA, USA), anti-CD45RA (PE; BD Biosciences), anti-CCR7 (PerCP/Cy 5.5; BioLegend, San Diego, CA, USA), anti-PD-1 (PE/Cy7; BioLegend), and Fixable Viability Dye (APC-Cy7; eBioscience). Both PBMC and decidual cells were stained with anti-CD3, anti-CD8, anti-CD45RA, anti-CCR7, and anti-PD-1 for 20 min on ice and then incubated for 5 min with Fixable Viability Dye to exclude dead cells. After staining, the cells were washed with PBS. Flow cytometric analysis and single cell sorting were performed using a FACSAria II flow cytometer (BD Biosciences). CD3^+^CD8^+^CD45RA^+^CCR7^+^ cells (naive CD8^+^ T cells; CD8^+^ N cells) and CD3^+^CD8^+^CD45RA^−^CCR7^−^ cells (effector memory CD8^+^ T cells; CD8^+^ EM cells) were single cell sorted into 96-well PCR plates (Supplementary Figures 1A,B). PD-1 expression in each cell was analyzed by the index sort method (Supplementary Figure 1C) (29).

### TCR Repertoire Analysis

TCR cDNAs were amplified from single cells using one-step RT-PCR, as previously described (30). All primers are listed in Supplementary Table 1. The contents of the PCR reaction mixture are listed in Supplementary Table 2. For the one-step RT-PCR, 5 μL of the RT-PCR mixture were added to each well containing a single CD8^+^ T cell. The program for the one-step RT-PCR was as follows: 40 min at 45°C for the RT reaction, 98°C for 1 min and 30 cycles of 98°C for 10 s, 52°C for 5 s, 72°C for 1 min. The amplification products were diluted 10-fold and 2 μL of each were added to 18 μL of the second PCR mixture. The PCR program for the second PCR cycle for TCRβ was as follows: 98°C for 1 min and 35 cycles of 98°C for 10 s, 52°C for 5 s, 72°C for 30 s. PCR products were electrophoresed to confirm their amplification (Supplementary Figure 2A) and then analyzed by direct sequencing. The TCR repertoire was analyzed with the IMGT/V-QUEST tool (http://www.imgt.org/). We defined CD8^+^ T cells in which the same TCRβ clonotype was detected two or more times as clonally expanded populations (clonal populations), and CD8^+^ T cells with a unique TCRβ clonotype as unique populations (Supplementary Figure 2B). The frequency of clonal populations and their PD-1 expression were compared in all groups.

### Statistical Analysis

Statistical analysis was performed using GraphPad Prism version 8 (GraphPad Software, San Diego, CA, USA). The differences between PBMC and decidua in each group were assessed using the Wilcoxon matched-pairs single rank test. A Mann-Whitney U test was performed to determine the differences between PBMC or decidual samples of different groups via two-group comparisons (control vs. 1st trimester or 3rd trimester normal pregnancy, 1st trimester vs. 3rd trimester normal pregnancy, 1st trimester normal pregnancy vs. miscarriage, and 3rd trimester normal pregnancy vs. preeclampsia). *p* < 0.05 were considered indicative of statistical significance (^*^p < 0.05; ^**^p < 0.01 in Wilcoxon matched-pairs single rank test; ^†^*p* < 0.05; ^†^*p* < 0.01 in Mann-Whitney U test; NS, not significant).

## Results

### CD8^+^ T Cell Phenotype in PBMC and Decidua

**Figure 1:**
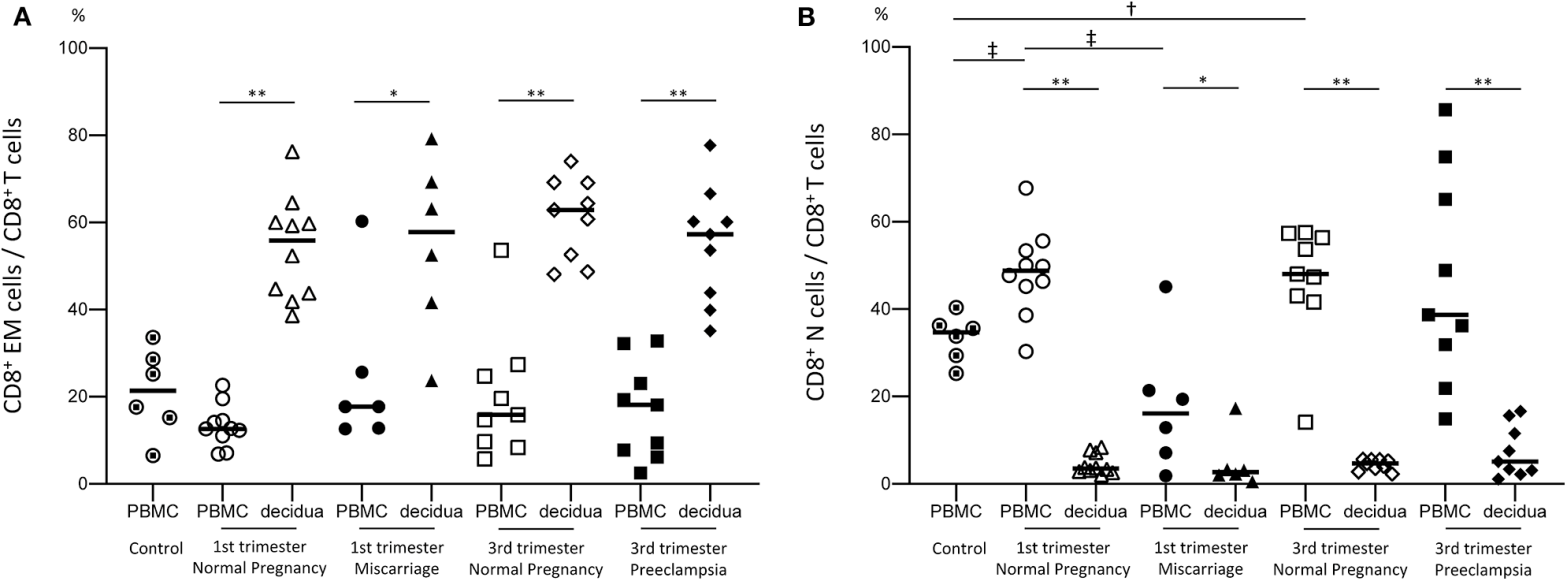
Proportion of CD8^+^ EM and CD8^+^ N cells among total CD8^+^ T cells. The proportion of CD8^+^ EM cells among CD8^+^ T cells **(A)** and that of CD8^+^ N cells among CD8^+^ T cells **(B)** are shown. Statistical analysis was performed using Wilcoxon matched-pairs single rank test (PBMC vs. decidua in each group); *p < 0.05; **p < 0.01. Mann-Whitney *U*-test (control vs. 1st or 3rd trimester normal pregnancy, 1st vs. 3rd trimester normal pregnancy, 1st trimester normal pregnancy vs. miscarriage, 3rd trimester normal pregnancy vs. preeclampsia); ^†^*p*<0.05; ^‡^*p*<0.01.

To examine functional differences between peripheral CD8^+^ T cells (pCD8^+^ T cells) and decidual CD8^+^ T cells (dCD8^+^ T cells), we compared the proportion of effector memory CD8^+^ T cells (CD8^+^ EM cells) and naive CD8^+^ T cells (CD8^+^ N cells) in the PBMC and decidua. A significantly higher number of CD8^+^ EM cells was observed in the decidua compared to the PBMC throughout the pregnancy period in normal pregnancy subjects, miscarriage cases, and preeclampsia cases (Figure 1A). In contrast, CD8^+^ N cells were significantly more abundant in the PBMC than in the decidua (Figure 1B). Therefore, dCD8^+^ T cells showed a distinct phenotype compared to pCD8^+^ T cells.

### Clonal Populations of CD8^+^ T Cells

**Figure 2:**
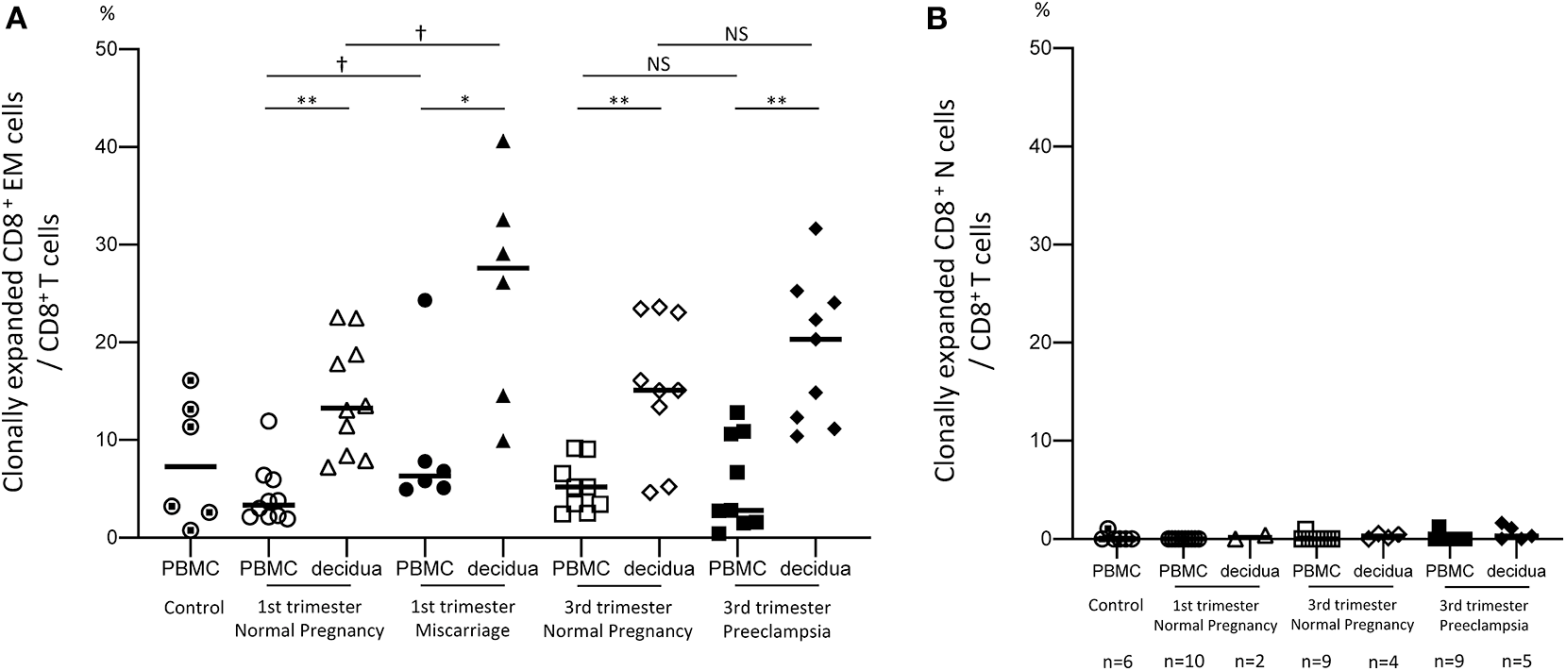
Clonal populations of CD8^+^ EM and CD8^+^ N cells among total CD8^+^ T cells. Clonally expanded CD8^+^ EM cells **(A)** and CD8^+^ N cells **(B)** among CD8^+^ T cells. Due to their small amount, CD8^+^ N cells could not be sorted in all samples. The number of cases analyzed in each group is shown under the horizontal axis. Statistical analysis was performed in CD8^+^ EM cells **(A)** by using Wilcoxon matched-pairs single rank test (PBMC vs. decidua in each group); *p < 0.05; **p < 0.01. Mann-Whitney U test (control vs. 1st or 3rd trimester normal pregnancy. 1st vs. 3rd trimester normal pregnancy, 1st trimester normal pregnancy vs. miscarriage, 3rd trimester normal pregnancy vs. preeclampsia); ^†^*p* < 0.05; NS not significant.

To verify our hypothesis that clonally expanded CD8^+^ T cells accumulate in the decidua, we analyzed the TCRβ clonotype of CD8^+^ T cells and their clonality ratio. As shown in Figure 2A and Supplementary Figure 3A, clonally expanded CD8^+^ T cell populations were observed both in the peripheral and decidual CD8^+^ EM cells. However, they were rarely detected in CD8^+^ N cells (Figure 2B, Supplementary Figure 3B). The clonality ratios of pCD8^+^ EM and dCD8^+^ EM cells among CD8^+^ EM cells were similar in early pregnant subjects, miscarriage cases, late pregnancy subjects, and preeclampsia cases (Supplementary Figure 3A). However, as shown in Figure 2A, the total amount of clonally expanded CD8^+^ EM cells among CD8^+^ T cells in the decidua was significantly higher than in the peripheral blood.

The total proportion of clonally expanded dCD8^+^ EM cells was significantly higher in miscarriage cases than in subjects with normal early pregnancy (*p* < 0.05). On the other hand, this population did not significantly differ between preeclampsia and normal late pregnancy (Figure 2A). These results demonstrated that dCD8^+^ EM cells are likely to recognize fetal or placental antigens in the decidua and are clonally expanded. An increased proportion of clonally expanded CD8^+^ EM cells was found to be associated with miscarriage.

### Common TCRβ Clonotype Between PBMC and Decidua

**Figure 3:**
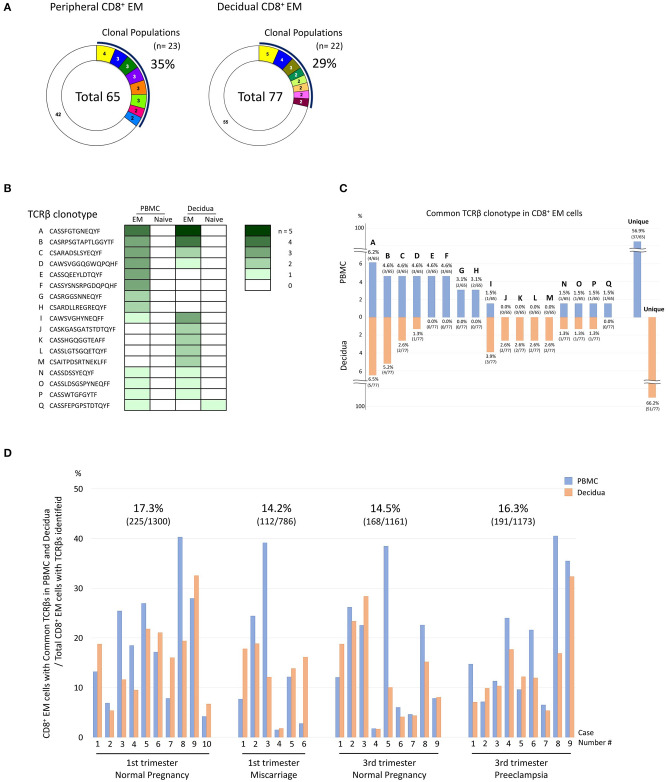
Comparison of TCRβ clonotype between PBMC and decidua. **(A–C)** A representative example of normal late pregnancy (case number #2). **(A)** Analysis of the TCRβ repertoire in peripheral CD8^+^ EM cells (pCD8^+^ EM cells) and decidual CD8^+^ N cells (dCD8^+^ N cells). Each pie slice in color indicates the T-cell population expressing the same clonotypic TCRβ. The numbers of CD8^+^ T cells expressing the same TCRβ are shown in the pie charts. The white slice in each pie chart indicates the T cell population with unique TCR. The number in the center of the pie charts is the total number of analyzed T cells. The proportion of clonal cells was calculated as follows: clonal population (%) = clonal CD8^+^ T cells/total CD8^+^ T cells analyzed. **(B)** The TCRβ clonotypes were compared between pCD8^+^ EM, pCD8^+^ N dCD8^+^ EM, and dCD8^+^ N cells. TCRβ clonotypes of clonally expanded CD8^+^ T cells in PBMC or decidua and/or TCRβ clonotypes in common between pCD8^+^ T and dCD8^+^ T cells are shown in a row (A to Q) (the color density reflects the number of clones). **(C)** Clones of clonally expanded CD8^+^ EM cells and/or clones that were shared between pCD8^+^ EM and dCD8^+^ EM cells are shown. The number above or under each bar is the percentage of the CD8^+^ EM cells with a particular TCR clone among total CD8^+^ EM cells with an identified TCRβ repertoire. **(D)** Comparison of the TCRβ clonotype between pCD8^+^ EM and dCD8^+^ EM cells in all cases. The blue bar indicates the CD8^+^ EM cell population with common TCRβ clonotype among total CD8^+^ EM cells with an identified TCRβ repertoire in PBMC; the orange bar indicates the corresponding population in the decidua. The total number of pCD8^+^ EM and dCD8^+^ EM cells expressing common TCRβ repertoires in all CD8^+^ EM cells of each group was calculated (the numbers above the bars in each group).

In each subject the TCRβ clonotype of CD8^+^ T cells was compared in paired PBMC and decidua to identify differential immunological functions (Figure 3). One representative sample of normal late pregnancy is shown in Figures 3A–C (case number #2). The proportion of clonally expanded CD8^+^ EM cells was comparable in PBMC and the decidua (Figure 3A). TCRβ clonotypes of clonally expanded CD8^+^ T cells and/or TCRβ clonotypes that are common to both pCD8^+^ T cells and dCD8^+^ T cells are shown in a row (A to Q) in Figure 3B (the different color density indicates the number of clones). Eight TCRβ clonotypes were shared between pCD8^+^ EM cells and dCD8^+^ EM cells (clonotypes A, B, C, D, I, N, O, and P in Figures 3B,C). Common TCRβ clonotypes between pCD8^+^ EM cells and dCD8^+^ EM cells were detected in all groups (Figure 3D). We calculated the ratios of pCD8^+^ EM cells and dCD8^+^ EM cells expressing common TCRβ clonotypes among the total CD8^+^ EM cells analyzed in each group. The ratios were comparable in all groups: 17.3% in 1st trimester normal pregnancy, 14.2% in miscarriage, 14.5% in 3rd trimester normal pregnancy, and 16.3% in preeclampsia (see the numbers above the bars in Figure 3D). These findings indicated that the immunological differences between normal pregnancy, miscarriage, and preeclampsia did not depend on the proportion of CD8^+^ EM cells expressing common TCRβ clonotypes in PBMC and the decidua.

### TCRβ Clonotype Comparison of the Serial Pregnancies

**Figure 4:**
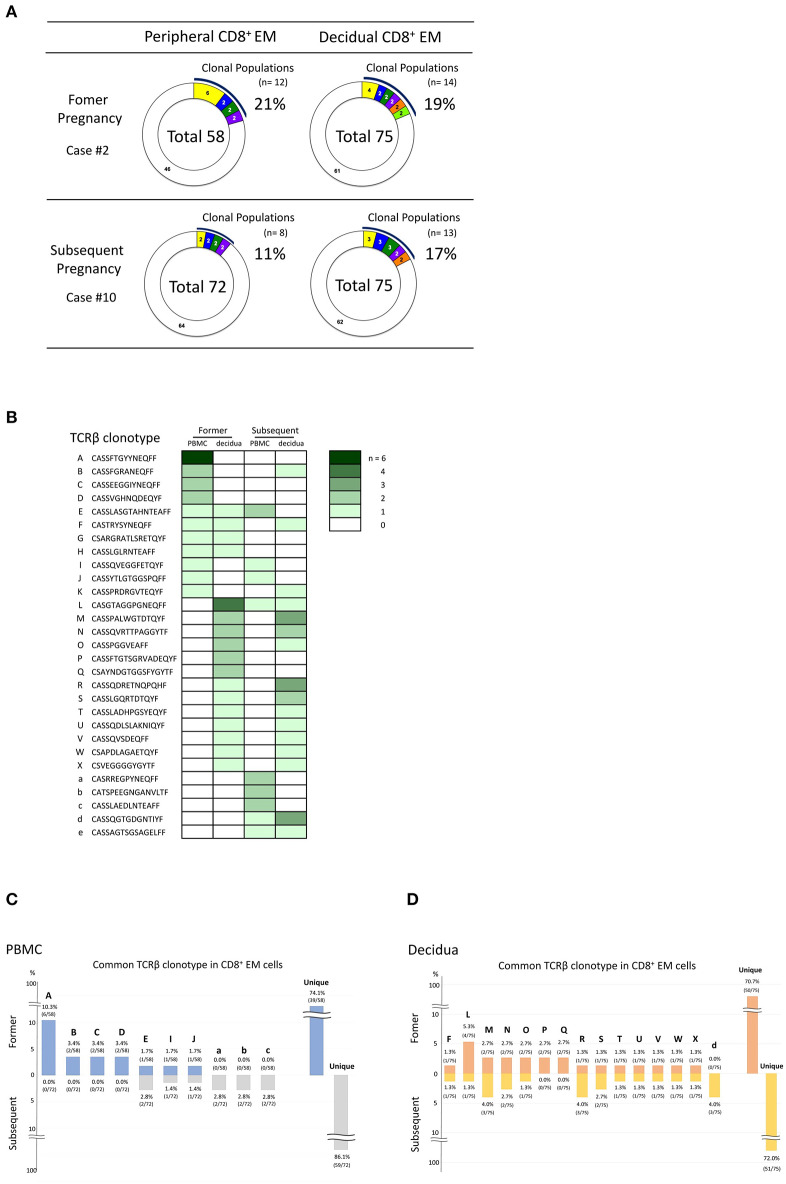
Comparison of the TCRβ clonotype during normal early pregnancy in consecutive pregnancies of the same subject (case numbers #2 and #10, obtained from the same patient). **(A)** Analysis of the TCRβ repertoire in pCD8^+^ EM and dCD8^+^ EM cells in different pregnancies of the same series. **(B)** TCRβ clonotype comparison in different pregnancies of the same series. TCRβ clonotypes of clonally expanded CD8^+^ EM cells in PBMC or decidua and/or TCRβ clonotypes in common between pCD8^+^ EM and dCD8^+^ EM cells are shown in a row (A to W, a to e) (the color density indicates the number of clones). Comparison of CD8^+^ EM TCRβ clonotype between two different pregnancies in PBMC **(C)** and the decidua **(D)**. The number above or under each bar is the percentage of CD8^+^ EM cells with a particular TCR clone among total CD8^+^ EM cells with an identified TCRβ repertoire.

If decidual CD8^+^ T cells recognize fetal antigens of paternal origin, CD8^+^ T cells with the same TCRβ clonotypes should be detected in different pregnancies of the same couple. To verify this hypothesis, we examined the TCRβ clonotypes of peripheral and decidual CD8^+^ EM cells in two pregnancies of the same subject with normal early pregnancy (Figure 4A). As shown in Figure 4B, the same TCRβ clonotypes were detected. Three TCRβ clonotypes (clone E, I, and J in Figure 4B) were shared by pCD8^+^ EM cell populations during the two pregnancies. Twelve TCRβ clonotypes (clone F, L, M, N, O, R, S, T, U, V, W, and X in Figure 4B) were found in dCD8^+^ EM cells from both pregnancies. Three TCRβ clonotypes (clone B, F, and K in Figure 4B) were shared between pCD8^+^ EM cells from the former pregnancy and dCD8^+^ EM cells from the subsequent pregnancy. Two TCRβ clonotypes (clone E and L in Figure 4B) were shared by dCD8^+^ EM cells from the former pregnancy and pCD8^+^ EM cells from the subsequent pregnancy; some clones were expanded in both pregnancies. CD8^+^ EM cells exhibiting the same TCRβ clonotypes in different pregnancies might be able to recognize fetal antigens.

### PD-1 Expression in CD8^+^ T Cells

**Figure 5:**
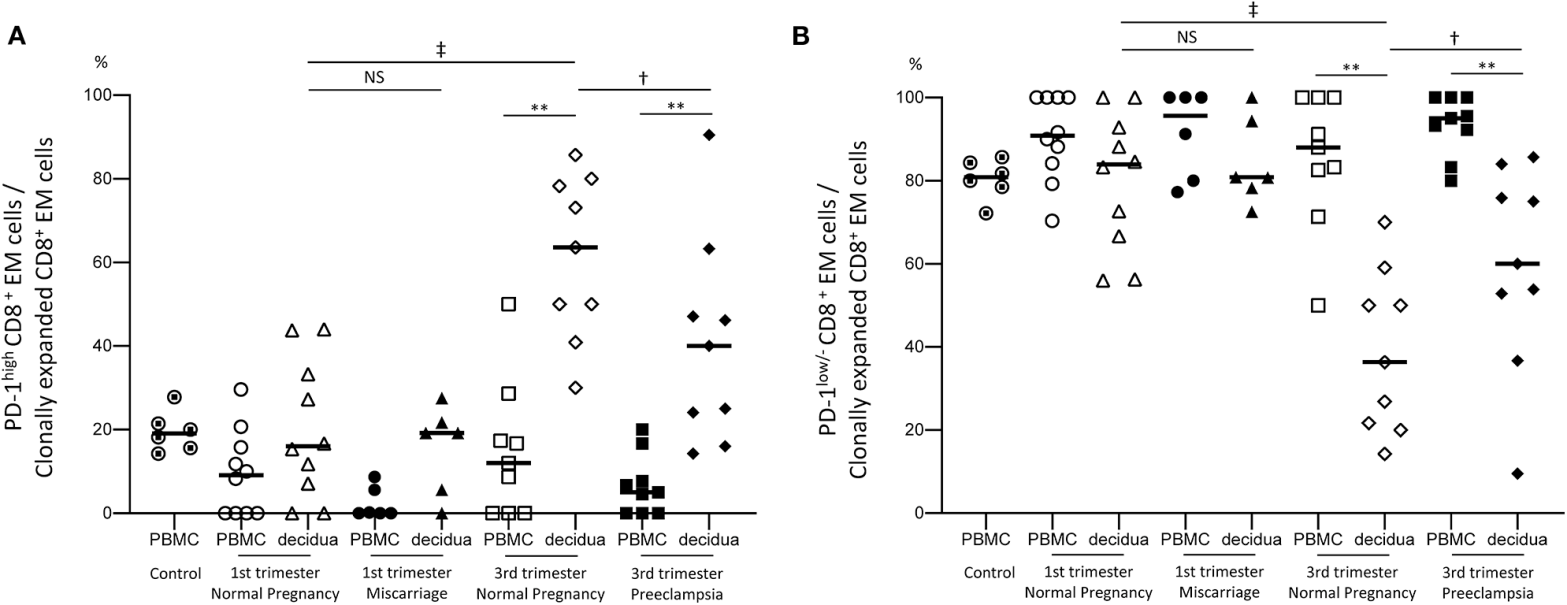
PD-1^high^ and PD-1^low/−^ CD8^+^ EM cell populations. **(A)** PD-1^high^ CD8^+^ EM cells among clonally expanded CD8^+^ EM cells. **(B)** PD-1^low/−^ CD8^+^ EM cells among clonally expanded CD8^+^ EM cells. Statistical analysis was performed using Wilcoxon matched-pairs single rank test (PBMC vs. decidua in each group); **p < 0.01. Mann-Whitney U-test (1st vs. 3rd trimester normal pregnancy, 1st trimester normal pregnancy vs. miscarriage, 3rd trimester normal pregnancy vs. preeclampsia); ^†^*p*<0.05; ^‡^*p*<0.01; NS, not significant.

Finally, the expression of PD-1 was analyzed in CD8^+^ T cells to identify immunological differences between normal pregnancy, miscarriage, and preeclampsia. Most CD8^+^ N cells were PD-1^low/−^ cells, both in the PBMC and the decidua (Supplementary Figure 4C). Among CD8^+^ EM cells, the size of the PD-1^low/−^ population was significantly lower in the decidua than in PBMC (Supplementary Figure 4A). In normal pregnancies, decidual PD-1^low/−^ CD8^+^ EM cells were significantly less abundant in late than in early pregnancy (*p* < 0.05) (Supplementary Figure 4A). When we focused on clonally expanded CD8^+^ EM cell populations, significant differences in decidual PD-1 expression were observed between normal pregnancy and preeclampsia cases during late pregnancy (Figures 5A,B). The population size of PD-1^high^ dCD8^+^ EM cells was significantly lower in preeclampsia than in normal late pregnancy (*p* < 0.05) (Figure 5A), indicating that the proportion of PD-1^low/−^ dCD8^+^ EM cells with high cytotoxic potential was increased in the clonal population of preeclampsia cases (Figure 5B). These findings indicated that dCD8^+^ EM cells had a higher level of PD-1 expression compared to pCD8^+^ EM cells, and that they begin to express PD-1 during pregnancy. An increased number of PD-1^low/−^ CD8^+^ EM cells would result in miscarriage, whereas, on the contrary, the downregulation of PD-1 in clonally expanded CD8^+^ EM cells would be associated with preeclampsia.

## Discussion

This study is the first to analyze the TCRβ repertoire and the expression of PD-1 in CD8^+^ T cells during pregnancy. The most abundant population of clonally expanded CD8^+^ EM cells was observed in the decidua. The proportion of clonally expanded CD8^+^ EM cells increased in cases of miscarriage, whereas PD-1 expression was downregulated in clonally expanded CD8^+^ EM cells in preeclampsia cases.

CD8^+^ T cells have a crucial role in immune tolerance at the feto-maternal interface. Previous studies have examined the distribution, phenotypes, gene, and cell surface protein expression, as well as functional properties of CD8^+^ T cells in both normal and complicated pregnancies (5, 7, 16, 17, 31, 32). Local expansion of highly differentiated decidual CD8^+^ T cells implies direct response to fetal-specific antigens. However, due to CD8^+^ T cell heterogeneity, it is unclear whether these decidual CD8^+^ T cells recognize fetal antigens. In recent studies, Zeng et al. performed a transcriptional analysis of paired pCD8^+^ T and dCD8^+^ T cell populations in the 1st trimester and revealed differences in gene regulation (33). Powell et al. identified fetal antigen-specific CD8^+^ T cells, both in the peripheral blood and the decidua, using HY-specific dextramers in humans (6, 34). The proportion of HY-specific CD8^+^ T cells was significantly increased in the decidua and most of them were CD8^+^ EM cells expressing the co-inhibitory molecule, PD-1 (6). These findings support the notion that CD8^+^ T cells recognizing fetal antigens exist but are functionally suppressed due to PD-1 expression at the feto-maternal interface. However, although minor populations of CD8^+^ T cells can be detected by the MHC-multimer approach, the extent of diversity of antigen-specific CD8^+^ T cells is still unclear. To our knowledge, this study is the first to examine the TCRβ repertoire of CD8^+^ EM cells and CD8^+^ N cells during pregnancy at the single-cell level. We also analyzed the clonotypes and PD-1 expression in these cells to identify tolerogenic differences between normal pregnancy, miscarriage, and preeclampsia.

Previous studies demonstrated that effector memory cells are the major subset of decidual CD8^+^ T cells, whereas naive cells predominate in the peripheral blood (5). Our results were consistent with previous studies (Figure 1) (5); we found that the proportion of CD8^+^ EM cells among decidual CD8^+^ T cells neither differed between normal early pregnancy and miscarriage, nor between normal late pregnancy and preeclampsia (Figure 1A). These findings indicated that pregnancy failure was not due to alterations in the proportion of CD8^+^ EM cells in early or late pregnancy.

Clonally expanded CD8^+^ T cells were observed in the effector memory subset but not in the naive subset (Figure 2), reflecting previous TCR stimulation and CD8^+^ T cell differentiation. A larger volume of clonally expanded CD8^+^ EM cells was observed in the decidua than in PBMC, and in late pregnancy compared to early pregnancy (Figure 2A). This suggested that CD8^+^ EM cells were expanded by antigen stimulation at the feto-maternal interface.

We observed common clonotypes between peripheral and decidual CD8^+^ EM cells (Figures 3B–D), reflecting the presence of effector memory T cell signatures in the systemic circulation. The ratio of CD8^+^ EM cells with common TCRβ clonotype was comparable in each group (Figure 3D), suggesting that the immunological differences between normal pregnancy, miscarriage, and preeclampsia did not depend on the proportion of CD8^+^ EM cells expressing the same TCRβ clonotype in PBMC and the decidua. CD8^+^ EM cells with common clonotypes in PBMC and the decidua might react with microchimeric fetal cells in the periphery (35–38).

If clonally expanded CD8^+^ EM cells recognize fetal antigens, the same TCRβ clonotype should be detected in different pregnancies of the same couple. Indeed, we found that some clonotypes were maintained across different pregnancies of the same patient (Figures 4B–D). Because of the high diversity of the CDR3 amino acid sequence in TCRβ, with a lower boundary of 2 × 10^7^ in young humans (27), these clonotype matches are unlikely to occur by chance. CD8^+^ EM cells with these TCR clonotypes might recognize the same fetal or placental antigens. In case of HLA-C mismatch between mother and fetus, and in the presence, in consecutive pregnancies, of the same paternal HLA-C, this could be recognized by CD8^+^ EM cells with the same TCRβ clonotype. Similarly, if the fetal sex of both pregnancies is male, the CD8^+^ EM cells might be HY-specific. Further studies are necessary to determine the antigens recognized by clonally expanded CD8^+^ EM cells. Another question to be addressed is whether clonally expanded CD8^+^ EM cells increase in parous women. As shown in Supplementary Figure 5, no differences in the size of clonally expanded CD8^+^ EM cell populations seemed to occur between nullipara and parous women. These findings suggested that the proportion of clonally expanded CD8^+^ EM cells did not increase in subsequent pregnancies.

The proportion of decidual PD-1^low/−^ CD8^+^ EM cells was larger in normal early pregnancy than in normal late pregnancy (Figure 5B); therefore, dCD8^+^ EM cells acquired PD-1 expression during late pregnancy. Wang et al. reported increased PD-1 expression in CD8^+^ T cells after co-culture with trophoblasts (7), suggesting that cell-cell interactions may induce PD-1 expression in CD8^+^ T cells at the feto-maternal interface. Other studies proposed the existence of CD8^+^ regulatory T cells with high PD-1 expression (39, 40). Therefore, the clonally expanded PD-1^high^ CD8^+^ EM cells detected in our study might be regulatory cells.

Clonally expanded CD8^+^ EM cells exhibited informative differences between normal pregnancy, miscarriage, and preeclampsia in terms of clonality and PD-1 expression. In the 1st trimester, a significant increase in the proportion of clonally expanded dCD8^+^ EM cells was observed in cases of miscarriage compared to normal pregnancy. In early pregnancy, most clonally expanded CD8^+^ EM cells were PD-1^low/−^. This suggested that an increase in antigen specific PD-1^low/−^ cytotoxic CD8^+^ EM cells at the feto-maternal interface might lead to miscarriage.

In contrast, in the 3rd trimester, the proportion of PD-1^low/−^ CD8^+^ EM cells among clonally expanded CD8^+^ EM cells was significantly increased in preeclampsia cases compared to normal pregnancy, despite a similar proportion of clonally expanded CD8^+^ EM cells (Figures 2A, 5B). Remarkably, the amount of PD-1^low/−^ CD8^+^ EM cells with unique TCRs did not differ between preeclampsia and normal pregnancy (Supplementary Figure 4B). These data suggest that antigen-specific CD8^+^ EM cells are less exhausted in preeclampsia.

We have previously reported that the total amount of decidual effector Treg cells is decreased in cases of miscarriage, whereas the size of clonal populations of decidual effector Treg cells is comparable in normal early pregnancy and miscarriage cases (24). On the other hand, the proportion of clonally expanded effector Treg cells in the decidua is lower in preeclampsia cases than in normal late pregnancy (24). Altogether, these observations indicate that the reduced proportion of non-specific decidual effector Treg cells and the increased proportion of clonally expanded PD-1^low/−^ cytotoxic dCD8^+^ T cells might lead to miscarriage in early pregnancy. In contrast, the decreased proportion of antigen-specific decidual effector Treg cells and the decreased expression of PD-1 in clonally expanded dCD8^+^ T cells might induce fetal rejection in preeclampsia. These results suggested that, in preeclampsia, antigen-specific tolerance was disrupted both in Treg cells and CD8^+^ T cells. This is in good accord with the epidemiology of human preeclampsia. The risk of preeclampsia increases in women at the first pregnancy following a partner change and after pregnancy intervals of more than 10 years (41–43). Increased risk of preeclampsia has also been reported in association with long-term condom usage and artificial insemination by donor, indicating that insufficient paternal antigen-specific tolerance mediated by seminal plasma priming may underlie preeclampsia (44–46). Pregnancy following oocyte donation, in which the fetus is completely allogeneic, associates with a significantly high risk of preeclampsia (45, 47). These epidemiological data demonstrate that the failure or lack of paternal antigen-specific tolerance may be responsible for preeclampsia. In addition, Barton et al. demonstrated that after the reencounter of fetal antigens by fetal antigen-specific CD8^+^ T cells, PD-1 expression was more effectively promoted in parous mice than virgin mice (48). The exposure to fetal antigens could promote PD-1 expression in CD8^+^ T cells, explaining the relatively high expression of PD-1 in late normal pregnancy. These epidemiological and experimental data suggest that the disruption of paternal antigen-specific tolerance in preeclampsia possibly affects PD-1 expression in CD8^+^ T cells, consistent with the PD-1 downregulation that we observed in clonally expanded CD8^+^ EM cells of preeclampsia cases.

Nevertheless, there are several limitations in this study. First, we assumed that clonally expanded CD8^+^ T cells might recognize fetal-specific antigens, but the target specificity of TCRs from CD8^+^ T cells has not been assessed. Second, although we assumed that clonally expanded dCD8^+^ EM cells were cytotoxic, this assumption was not verified. Third, we focused on PD-1 expression, while alterations of other co-inhibitory molecules and cytokine expression have been reported in miscarriage and preeclampsia. Extensive analysis of these factors as well as the TCR repertoire may help understand the immunological differences between normal pregnancy, miscarriage, and preeclampsia. An additional limitation is that the detrimental immune reactivity of CD8^+^ T cells observed in miscarriage and preeclampsia may be partly due to different timing of sample collection (at 6–8 gestational weeks in miscarriage, and at 32–39 gestational weeks in preeclampsia), albeit this problem cannot be overcome.

In conclusion, CD8^+^ EM cells might recognize some antigens at the feto-maternal interface, which are clonally expanded in the decidua. Clonally expanded dCD8^+^ EM cells expressed PD-1 on their surface during late pregnancy, although most of them did not express PD-1 during early pregnancy. The total proportion of PD-1^low/−^ clonally expanded CD8^+^ EM cells increased in both miscarriage and preeclampsia cases, but the mechanisms behind this phenomenon were distinct. In miscarriage cases, the proportion of clonally expanded CD8^+^ EM cells increased. On the other hand, in preeclampsia, clonally expanded dCD8^+^ EM cells exhibited low PD-1 expression. Based on the results of this and our former study, we can conclude that, in miscarriage, the total proportion of decidual effector Treg cells decreased, while that of clonally expanded dCD8^+^ EM cells increased. Moreover, in preeclampsia, the proportion of clonally expanded decidual effector Treg cells decreased and PD-1 expression was downregulated in the clonally expanded dCD8^+^ EM cells. Thus, the recognition of fetal antigens by clonally expanded Treg cells and CD8^+^ EM cells would easily induce fetal rejection. In future studies, we will attempt to clarify which antigens are recognized by clonally expanded TCRβ.

## Data Availability Statement

The datasets presented in this article are not readily available because the ethics review committee of the University of Toyama, which approved our protocol, did not give permission for data sharing. Requests to access the datasets should be directed to s30saito@med.u-toyama.ac.jp.

## Ethics Statement

This study was carried out in accordance with the recommendations of the Ethical Guidelines for Medical and Health Research Involving Human Subjects, the Ministry of Health, Labor and Welfare, Japan. The protocol was approved by the ethics review committee of the University of Toyama (Rin 28- 144). All subjects gave written informed consent in accordance with the Declaration of Helsinki.

## Author Contributions

SS and HK: conception and design. KM, ST, and AU: acquiring and processing samples. KM, EK, HH, and KT: execution of experiment. KM and ST: analysis of data. KM, ST, EK, HH, HK, and SS: interpretation of data. KM, ST, and SS: drafting manuscript. ST, EK, HH, TS, AN, HK, and SS: revision of the manuscript for important intellectual content.

## Conflict of Interest

The authors declare that the research was conducted in the absence of any commercial or financial relationships that could be construed as a potential conflict of interest.

## References

[1] Saito S Nishikawa K Morii T Narita N Enomoto M Ichijo M. Expression of activation antigens CD69, HLA-DR, interleukin-2 receptor-alpha (IL-2R alpha) and IL-2R beta on T cells of human decidua at an early stage of pregnancy. Immunology. (1992) 75:710–2. 1592443 PMC1384855

[2] Tilburgs T Scherjon SA van der Mast BJ Haasnoot GW Versteeg VDV-MM Roelen DL. Fetal-maternal HLA-C mismatch is associated with decidual T cell activation and induction of functional T regulatory cells. J Reprod Immunol. (2009) 82:148–57. 10.1016/j.jri.2009.05.003 19631389

[3] Aluvihare VR Kallikourdis M Betz AG. Regulatory T cells mediate maternal tolerance to the fetus. Nat Immunol. (2004) 5:266–71. 10.1038/ni1037 14758358

[4] Sasaki Y Sakai M Miyazaki S Higuma S Shiozaki A Saito S. Decidual and peripheral blood CD4+CD25+ regulatory T cells in early pregnancy subjects and spontaneous abortion cases. Mol Hum Reprod. (2004) 10:347–53. 10.1093/molehr/gah044 14997000

[5] Tilburgs T Schonkeren D Eikmans M Nagtzaam NM Datema G Swings GM. Human decidual tissue contains differentiated CD8+ effector-memory T cells with unique properties. J Immunol. (2010) 185:4470–7. 10.4049/jimmunol.0903597 20817873

[6] Powell RM Lissauer D Tamblyn J Beggs A Cox P Moss P Decidual T cells exhibit a highly differentiated phenotype and demonstrate potential fetal specificity and a strong transcriptional response to IFN. J Immunol. (2017) 199:3406–17. 10.4049/jimmunol.1700114 28986438 PMC5679367

[7] Wang SC Li YH Piao HL Hong XW Zhang D Xu YY. PD-1 and Tim-3 pathways are associated with regulatory CD8+ T-cell function in decidua and maintenance of normal pregnancy. Cell Death Dis. (2015) 6:e1738. 10.1038/cddis.2015.112 25950468 PMC4669692

[8] van der Zwan A Bi K Norwitz ER Crespo AC Claas FHJ Strominger JL. Mixed signature of activation and dysfunction allows human decidual CD8(+) T cells to provide both tolerance and immunity. Proc Natl Acad Sci USA. (2018) 115:385–90. 10.1073/pnas.1713957115 29259116 PMC5777048

[9] Taglauer E.S. Trikhacheva AS Slusser JG Petroff MG. Expression and function of PDCD1 at the human maternal-fetal interface. Biol Reprod. (2008) 79:562–9. 10.1095/biolreprod.107.066324 18550794 PMC2688813

[10] Meggyes M Miko E Szigeti B Farkas N Szereday L. The importance of the PD-1/PD-L1 pathway at the maternal-fetal interface. BMC Pregn Childbirth. (2019) 19:74. 10.1186/s12884-019-2218-6 30782114 PMC6381664

[11] Petroff MG Chen L Phillips TA Azzola D Sedlmayr P Hunt. B7 family molecules are favorably positioned at the human maternal-fetal interface. Biol Reprod. (2003) 68:1496–504. 10.1095/biolreprod.102.010058 12606489

[12] Guleria I Khosroshahi A Ansari MJ Habicht A Azuma M Yagita H. A critical role for the programmed death ligand 1 in fetomaternal tolerance. J Exp Med. (2005) 202:231–7. 10.1084/jem.20050019 16027236 PMC2213002

[13] Nagamatsu T Schust DJ Sugimoto J Barrier BF. Human decidual stromal cells suppress cytokine secretion by allogenic CD4+ T cells via PD-1 ligand interactions. Hum Reprod. (2009) 24:3160–71. 10.1093/humrep/dep308 19729380

[14] Arck PC Hecher K. Fetomaternal immune cross-talk and its consequences for maternal and offspring's health. Nat Med. (2013) 19:548–56. 10.1038/nm.3160 23652115

[15] Vento-Tormo R Efremova M Botting RA Turco MY Vento-Tormo M Meyer KB. Single-cell reconstruction of the early maternal-fetal interface in humans. Nature. (2018) 563:347–53. 10.1038/s41586-018-0698-6 30429548 PMC7612850

[16] Taglauer ES Yankee TM Petroff MG. Maternal PD-1 regulates accumulation of fetal antigen-specific CD8+ T cells in pregnancy. J Reprod Immunol. (2009) 80:12–21. 10.1016/j.jri.2008.12.001 19368976 PMC2764286

[17] Tilburgs T Strominger JL. CD8+ effector T cells at the fetal-maternal interface balancing fetal tolerance and antiviral immunity. Am J Reprod Immunol. (2013) 69:395–407. 10.1111/aji.12094 23432707 PMC3711858

[18] Ramhorst R Garcia V Agriello E Corigliano A Etchepareborda E Irigoyen M. Intracellular expression of CD69 in endometrial and peripheral T cells represents a useful marker in women with recurrent miscarriage: modulation after allogeneic leukocyte immunotherapy. Am J Reprod Immunol. (2003) 49:149–58. 10.1034/j.1600-0897.2003.00021.x 12797521

[19] Yang H Qiu L Chen G Ye Z Lu C Lin Q. Proportional change of CD4+CD25+ regulatory T cells in decidua and peripheral blood in unexplained recurrent spontaneous abortion patients. Fertil Steril. (2008) 89:656–61. 10.1016/j.fertnstert.2007.03.037 17543960

[20] Mei S Tan J Chen H Chen Y Zhang J. Changes of CD4+CD25high regulatory T cells and FOXP3 expression in unexplained recurrent spontaneous abortion patients. Fertil Steril. (2010) 94:2244–7. 10.1016/j.fertnstert.2009.11.020 20056219

[21] Sasaki Y Darmochwal-Kolarz D Suzuki D Sakai M Ito M Shima T. Proportion of peripheral blood and decidual CD4(+) CD25(bright) regulatory T cells in pre-eclampsia. Clin Exp Immunol. (2007) 149:139–45. 10.1111/j.1365-2249.2007.03397.x 17459078 PMC1942015

[22] Toldi G Svec P Vasarhelyi B Meszaros G Rigo J Tulassay T. Decreased number of FoxP3+ regulatory T cells in preeclampsia. Acta Obstet Gynecol Scand. (2008) 87:1229–33. 10.1080/00016340802389470 19016357

[23] Santner-Nanan B Peek MJ Khanam R Richarts L Zhu E Fazekas de St Groth B. Systemic increase in the ratio between Foxp3+ and IL-17-producing CD4+ T cells in healthy pregnancy but not in preeclampsia. J Immunol. (2009) 183:7023–30. 10.4049/jimmunol.0901154 19915051

[24] Tsuda S Zhang X Hamana H Shima T Ushijima A Tsuda K. Clonally expanded decidual effector regulatory T cells increase in late gestation of normal pregnancy but not in preeclampsia in humans. Front Immunol. (2018) 9:1934. 10.3389/fimmu.2018.01934 30197648 PMC6118230

[25] Tafuri A Alferink J Moller P Hammerling GJ Arnold B. T cell awareness of paternal alloantigens during pregnancy. Science. (1995) 270:630–3. 10.1126/science.270.5236.630 7570020

[26] Moldenhauer LM Hayball JD Robertson SA. Utilising T cell receptor transgenic mice to define mechanisms of maternal T cell tolerance in pregnancy. J Reprod Immunol. (2010) 87:1–13. 10.1016/j.jri.2010.05.007 20615552

[27] Qi Q Liu Y Cheng Y Glanville J Zhang D Lee JY. Diversity and clonal selection in the human T-cell repertoire. Proc Natl Acad Sci USA. (2014) 111:13139–44. 10.1073/pnas.1409155111 25157137 PMC4246948

[28] Brown MA Magee LA Kenny LC Karumanchi SA McCarthy FP Saito S. Hypertensive disorders of pregnancy: ISSHP classification diagnosis and management recommendations for international practice. Hypertension. (2018) 72:24–43. 10.1161/HYPERTENSIONAHA.117.10803 29899139

[29] Penter L Dietze K Bullinger L Westermann J Rahn HP Hansmann L. FACS single cell index sorting is highly reliable and determines immune phenotypes of clonally expanded T cells. Eur J Immunol. (2018) 48:1248–50. 10.1002/eji.201847507 29537492

[30] Hamana H Shitaoka K Kishi H Ozawa T Muraguchi A. A novel rapid and efficient method of cloning functional antigen-specific T-cell receptors from single human and mouse T-cells. Biochem Biophys Res Commun. (2016) 474:709–14. 10.1016/j.bbrc.2016.05.015 27155153

[31] Shao L Jacobs AR Johnson VV Mayer L. Activation of CD8+ regulatory T cells by human placental trophoblasts. J Immunol. (2005) 174:39–7547. 10.4049/jimmunol.174.12.7539 15944253

[32] Tilburgs T Roelen DL van der Mast BJ van Schip JJ Kleijburg C de Groot-Swings GM. Differential distribution of CD4(+)CD25(bright) and CD8(+)CD28(-) T-cells in decidua and maternal blood during human pregnancy. Placenta. (2006) 27(Suppl. A):S47–53. 10.1016/j.placenta.2005.11.008 16442616

[33] Zeng W Liu X Liu Z Zheng Y Yu T Fu S. Deep surveying of the transcriptional and alternative splicing signatures for decidual cd8(+) T cells at the first trimester of human healthy pregnancy. Front Immunol. (2018) 9:937. 10.3389/fimmu.2018.00937 29780389 PMC5946033

[34] Lissauer D Piper K Goodyear O Kilby MD Moss PA. Fetal-specific CD8+ cytotoxic T cell responses develop during normal human pregnancy and exhibit broad functional capacity. J Immunol. (2012) 189:1072–80. 10.4049/jimmunol.1200544 22685312

[35] Bianchi DW Zickwolf GK Weil GJ Sylvester S DeMaria MA. Male fetal progenitor cells persist in maternal blood for as long as 27 years postpartum. Proc Natl Acad Sci USA. (1996) 93:705–8. 10.1073/pnas.93.2.705 8570620 PMC40117

[36] Lo YM Lo ES Watson N Noakes L Sargent IL Thilaganathan B. Two-way cell traffic between mother and fetus: biologic and clinical implications. Blood. (1996) 88:4390–95. 10.1182/blood.V88.11.4390.4390 8943877

[37] O'Donoghue K Chan J de la Fuente J Kennea N Sandison A Anderson JR. Microchimerism in female bone marrow and bone decades after fetal mesenchymal stem-cell trafficking in pregnancy. Lancet. (2004) 364:179–82. 10.1016/S0140-6736(04)16631-2 15246731

[38] Nelson J. Your cells are my cells. Sci Am. (2008) 298:64–71. 10.1038/scientificamerican0408-64 18376674

[39] Arruvito L Payaslian F Baz P Podhorzer A Billordo A Pandolfi J. Identification and clinical relevance of naturally occurring human CD8+HLA-DR+ regulatory T cells. J Immunol. (2014) 193:4469–76. 10.4049/jimmunol.1401490 25261474

[40] Machicote A Belen S Baz P Billordo LA Fainboim L. Human CD8(+)HLA-DR(+) regulatory T cells similarly to classical CD4(+)Foxp3(+) cells suppress immune responses via PD-1/PD-L1 Axis. Front Immunol. (2018) 9:2788. 10.3389/fimmu.2018.02788 30555473 PMC6281883

[41] Robillard PY Hulsey TC Alexander GR Keenan A de Caunes F Papiernik E. Paternity patterns and risk of preeclampsia in the last pregnancy in multiparae. J Reprod Immunol. (1993) 24:1–12. 10.1016/0165-0378(93)90032-D 8350302

[42] Trupin LS Simon LP Eskenazi B. Change in paternity: a risk factor for preeclampsia in multiparas. Epidemiology. (1996) 7:240–4. 10.1097/00001648-199605000-00004 8728435

[43] Skjaerven R Wilcox AJ Lie RT. The interval between pregnancies and the risk of preeclampsia. N Engl J Med. (2002) 346:33–8. 10.1056/NEJMoa011379 11778000

[44] Klonoff-Cohen HS Savitz DA Cefalo RC McCann MF. An epidemiologic study of contraception and preeclampsia. JAMA. (1989) 262:3143–7. 10.1001/jama.262.22.3143 2810672

[45] Salha O Sharma V Dada T Nugent D Rutherford AJ Tomlinson AJ. The influence of donated gametes on the incidence of hypertensive disorders of pregnancy. Hum Reprod. (1999) 14:2268–73. 10.1093/humrep/14.9.2268 10469693

[46] Koelman CA Coumans AB Nijman HW Doxiadis II Dekker GA Claas FH. Correlation between oral sex and a low incidence of preeclampsia: a role for soluble HLA in seminal fluid? J Reprod Immunol. (2000) 46:155–66. 10.1016/S0165-0378(99)00062-5 10706945

[47] Nakabayashi Y Nakashima A Yoshino O Shima T Shiozaki A Adachi T Impairment of the accumulation of decidual T cells NK cells and monocytes and the poor vascular remodeling of spiral arteries were observed in oocyte donation cases regardless of the presence or absence of preeclampsia. J Reprod Immunol. (2016) 114:65–74. 10.1016/j.jri.2015.07.005 26282090

[48] Barton BM Xu R Wherry EJ Porrett PM. Pregnancy promotes tolerance to future offspring by programming selective dysfunction in long-lived maternal T cells. J Leukoc Biol. (2017) 101:975–87. 10.1189/jlb.1A0316-135R 27810945 PMC12040000

