# Supplementary material for: Analysis of TCR Repertoire and PD-1 Expression in Decidual and Peripheral CD8+ T Cells Reveals Distinct Immune Mechanisms in Miscarriage and Preeclampsia
Source: Front Immunol. 2020 Jun 3;11:1082. doi: 10.3389/fimmu.2020.01082 (PMC7283903; doi:10.3389/fimmu.2020.01082)
Supplement: Supplementary file 3 [file Data_Sheet_1.pdf]

Supplementary Material

Supplementary Tables

Supplementary Table 1. Primers for single-cell RT-PCR

Supplementary Table 2. Contents of single-cell RT-PCR reaction mix.

Supplementary Figures

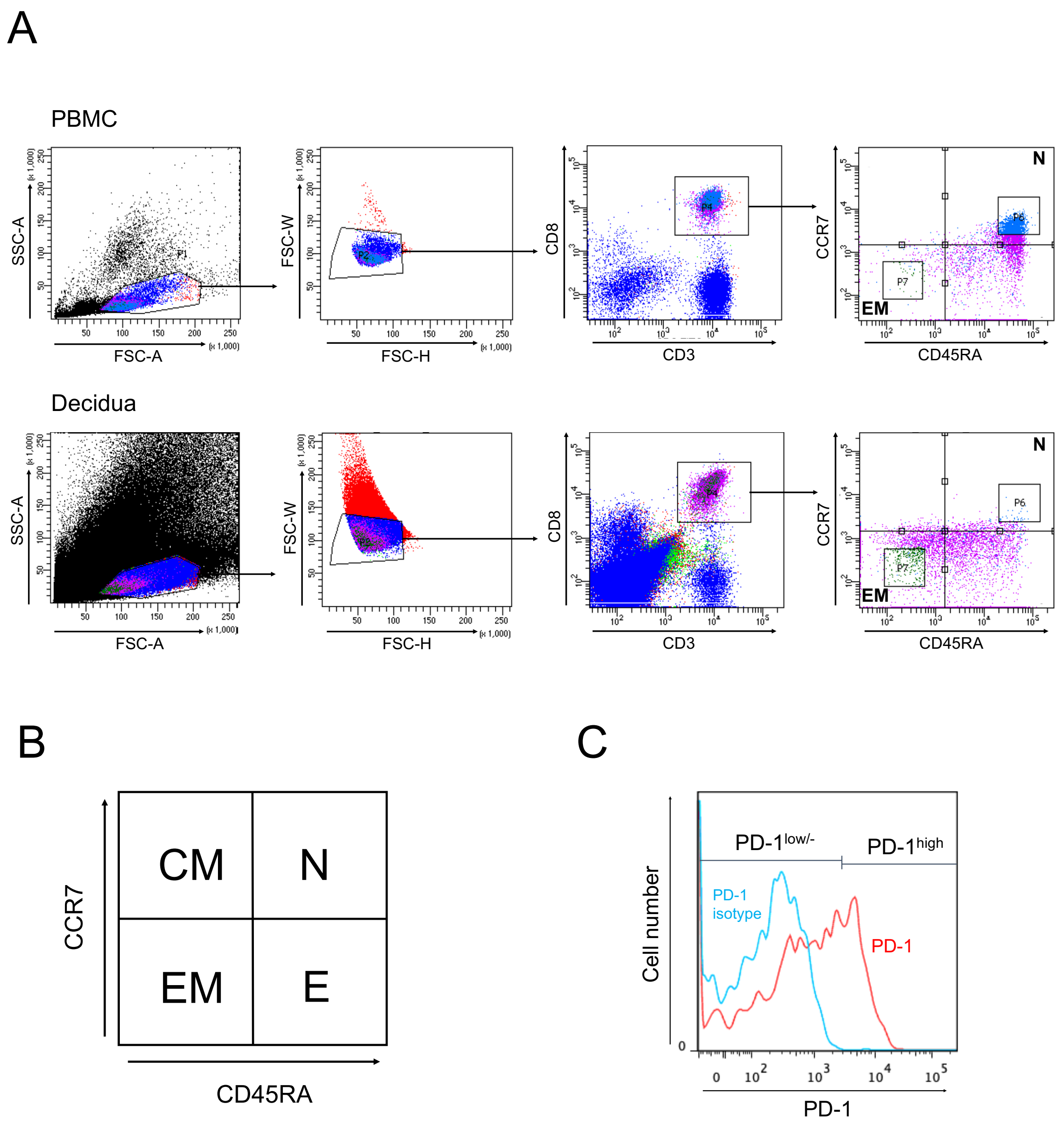

Supplementary Figure 1. Gating strategy to obtain $CD3^+CD8^+CD45RA^-CCR7^-$ effector memory T cells ($CD8^+$ EM cells) and $CD3^+CD8^+CD45RA^+CCR7^+$ naive T cells ($CD8^+$ N cells). **(A)** Both $CD8^+$ EM cells and $CD8^+$ N cells were single-cell sorted from PBMC (upper) and decidua (lower). **(B)** Classification of memory phenotype. N: naive, E: effector, EM: effector memory, CM: central memory. **(C)** PD-1 expression was distinguished into PD-1^{high} and PD-$1^{low/-}$ as shown in Supplementary Figure 1C.

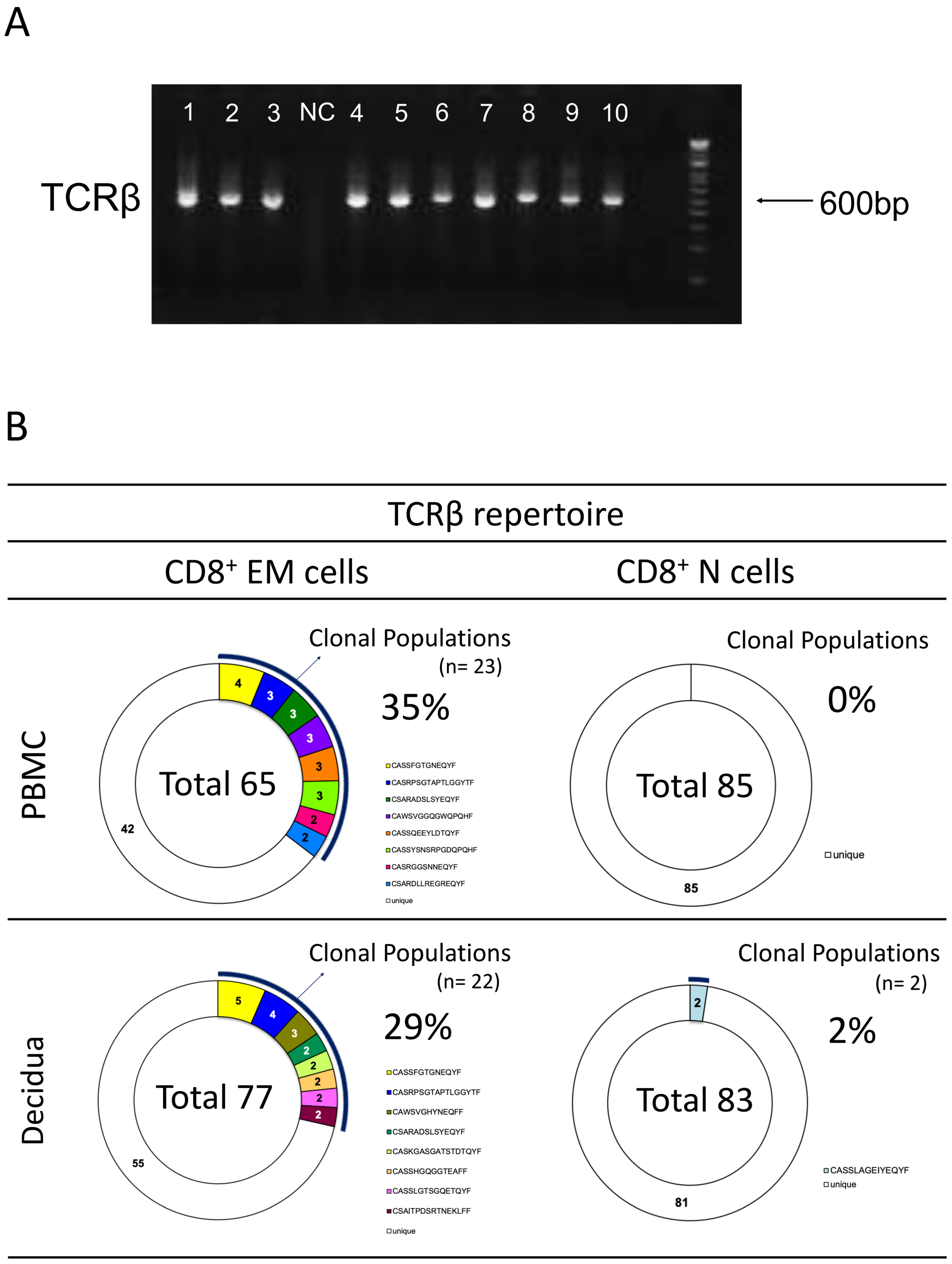

Supplementary Figure 2. TCRβ repertoire analysis in effector memory CD8$^+$ T cells and naive CD8$^+$ T cells. **(A)** Expression of TCRβ cDNAs after RT-PCR amplification and electrophoresis. **(B)** Representative data of the TCRβ repertoire analysis in CD8$^+$ EM cells and CD8$^+$ N cells from PBMCs and decidua in 3rd-trimester normal pregnancy. Each colored pie slice indicates T-cell populations expressing the same clonotypic TCRβ. The numbers of CD8$^+$ T cells expressing the same TCRβ clonotype are shown in the pie charts. The white slice in each pie chart indicates the T cell population with unique TCR. The number in the center of the pie charts is the total number of analyzed T cells. The proportion of clonal population was calculated as follows: clonal population (%) = clonal CD8$^+$ T cells / total CD8$^+$ T cells analyzed.

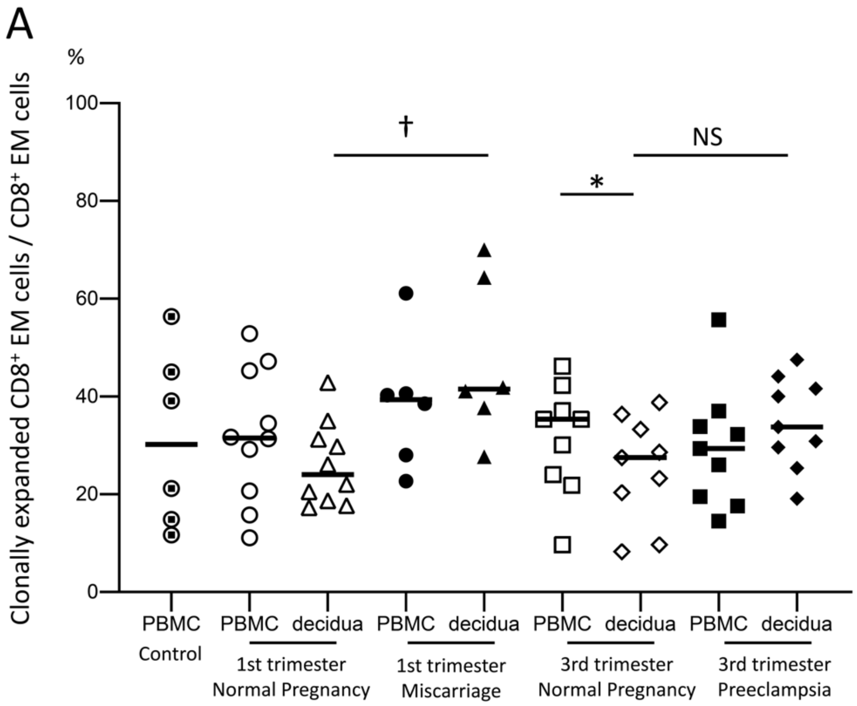

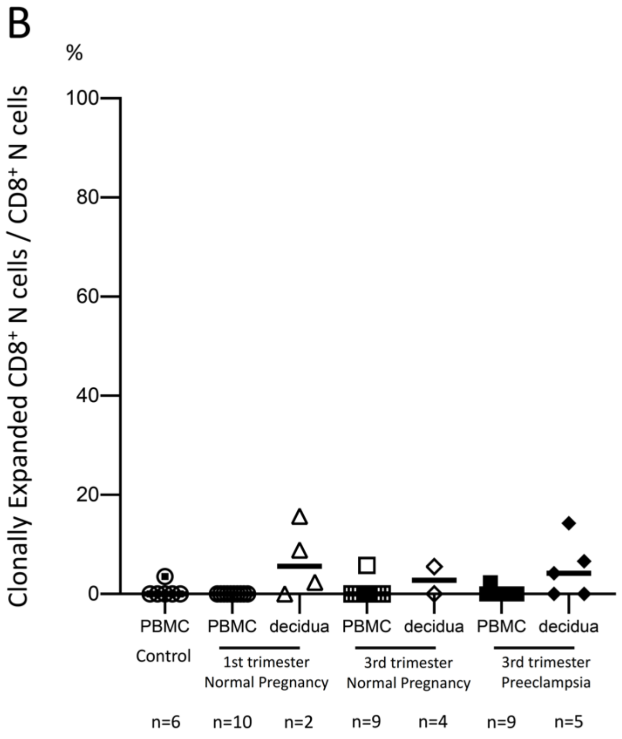

Supplementary Figure 3. Clonally expanded populations of effector memory $CD8^+$ T cells among $CD8^+$ EM cells (A) and of naive $CD8^+$ T cells in $CD8^+$ N cells (B). Wilcoxon matched-pairs single rank test (PBMC vs decidua in each group); p <0.05; *. Mann-Whitney U test (1st vs 3rd normal pregnancy, 1st normal pregnancy vs miscarriage, 3rd normal pregnancy vs preeclampsia); †, p<0.05; NS, not significant.

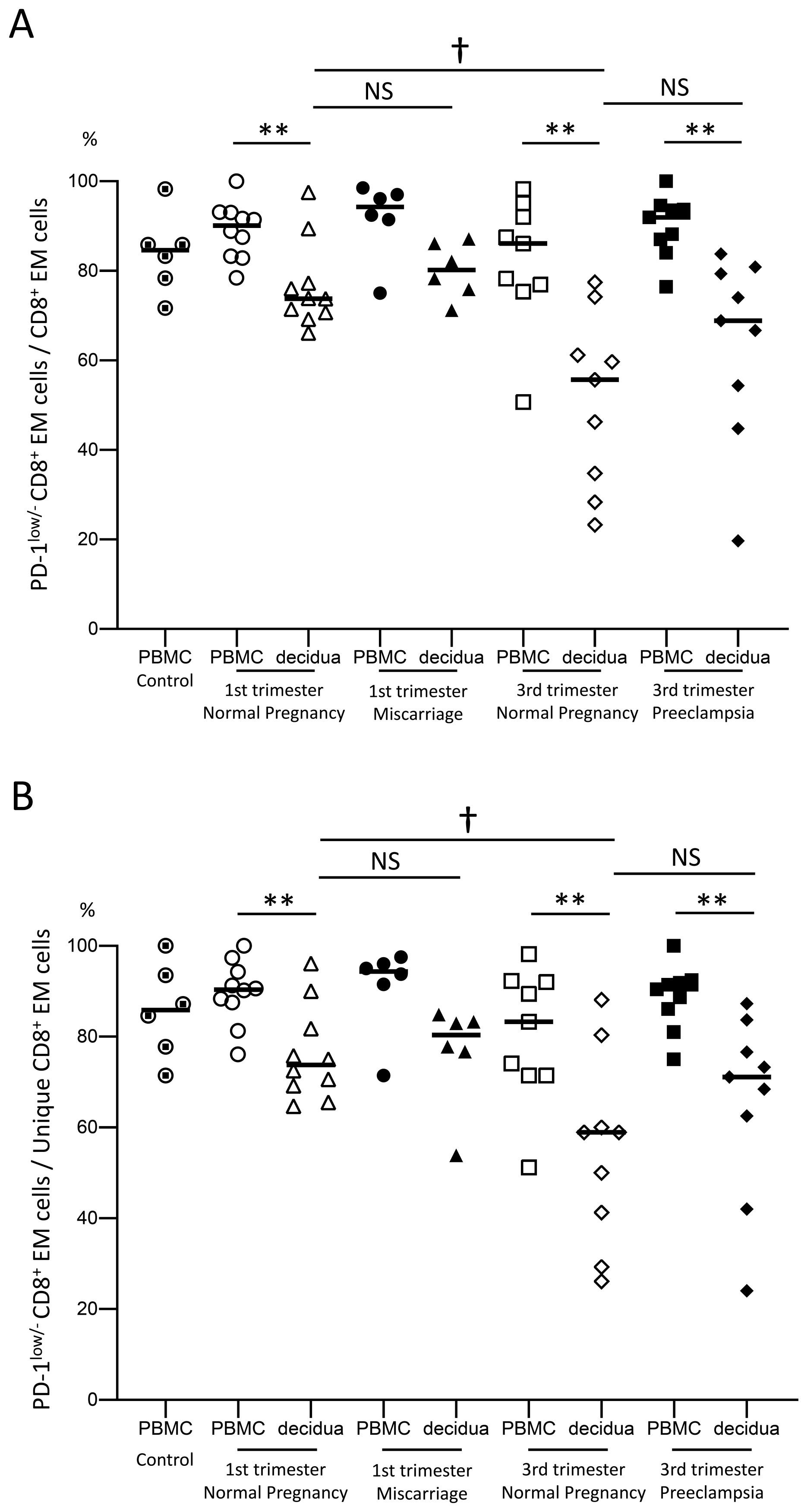
A
†
NS
NS
**
**
**
%
PD-1low/- CD8+ EM cells / CD8+ EM cells
100
80
60
40
20
0
PBMC
Control
PBMC decidua
1st trimester
Normal Pregnancy
PBMC decidua
1st trimester
Miscarriage
PBMC decidua
3rd trimester
Normal Pregnancy
PBMC decidua
3rd trimester
Preeclampsia
B
†
NS
NS
**
**
**
%
PD-1low/- CD8+ EM cells / Unique CD8+ EM cells
100
80
60
40
20
0
PBMC
Control
PBMC decidua
1st trimester
Normal Pregnancy
PBMC decidua
1st trimester
Miscarriage
PBMC decidua
3rd trimester
Normal Pregnancy
PBMC decidua
3rd trimester
Preeclampsia

C

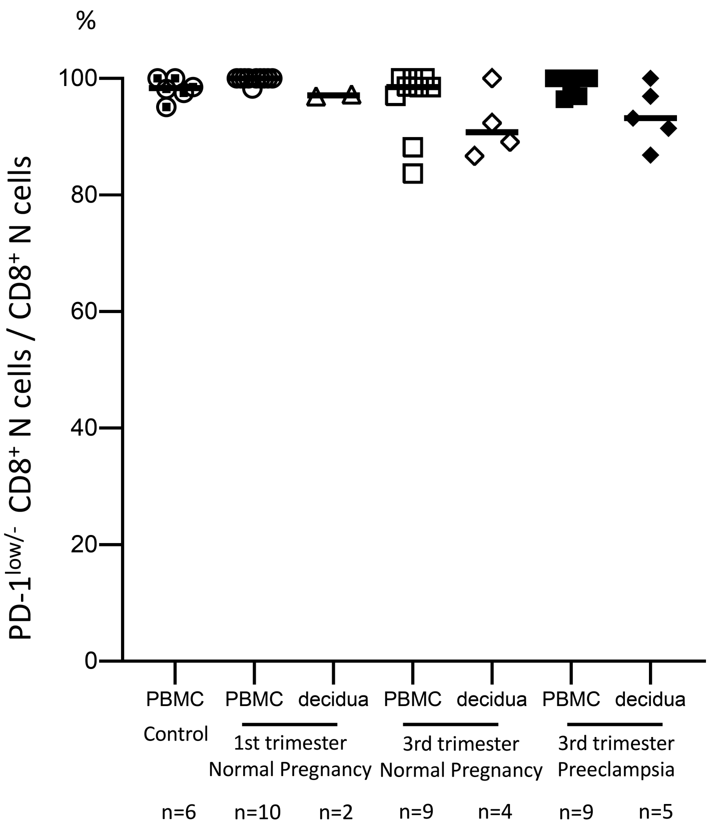

Supplementary Figure 4. PD-1$^{low/-}$ CD8^{+} T cell population. PD-1$^{low/-}$ CD8^{+} EM cell populations among CD8^{+} EM cells **(A)** and among CD8^{+} EM cells with unique TCRβ clonotype **(B)**. **(C)** PD-1$^{low/-}$ CD8^{+} N cells among CD8^{+} N cells. Wilcoxon matched-pairs single rank test (PBMC vs decidua in each group); **, p<0.01. Mann-Whitney U test (1st vs 3rd normal pregnancy, 1st normal pregnancy vs miscarriage, 3rd normal pregnancy vs preeclampsia); †, p<0.05; NS, not significant.

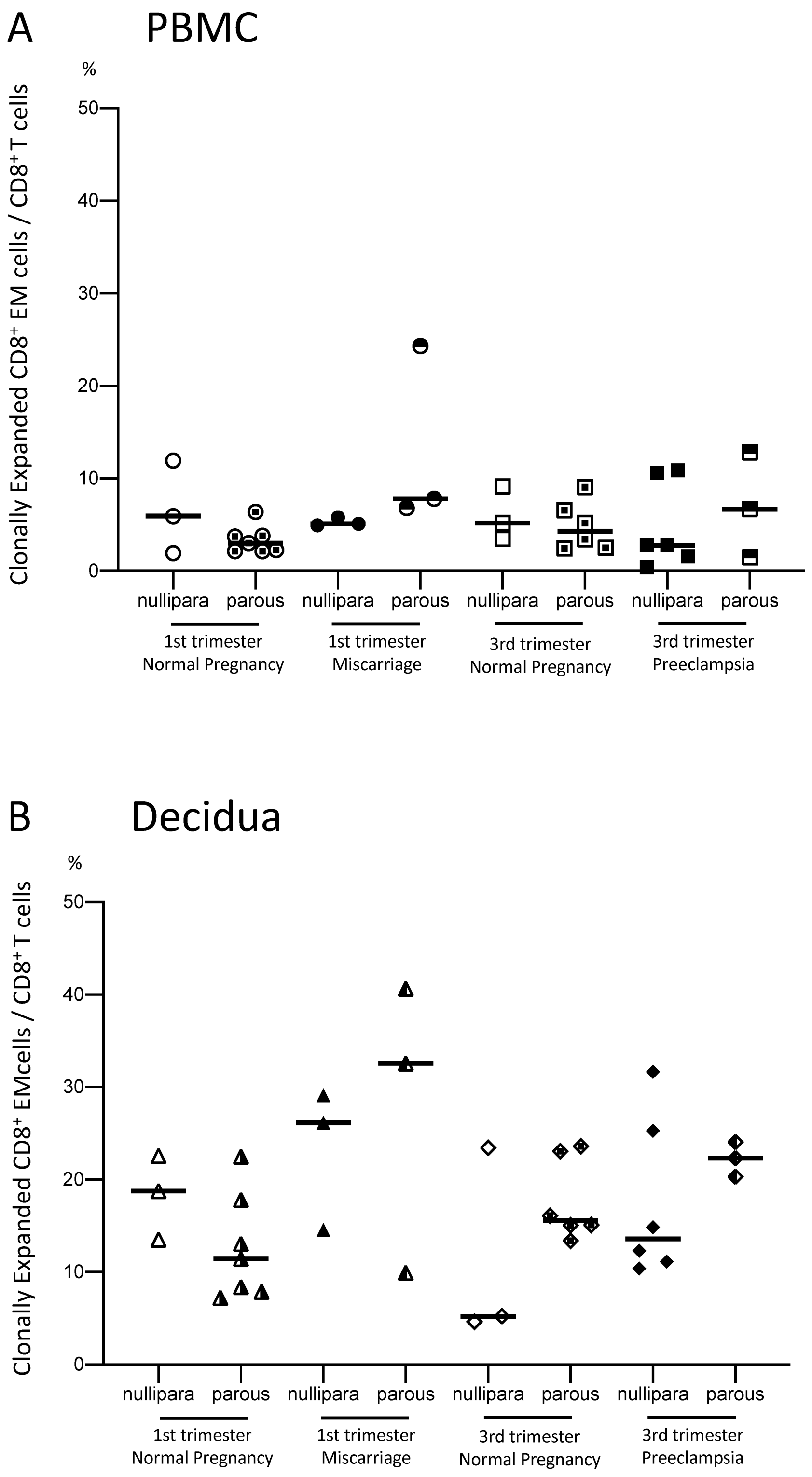

Supplementary Figure 5. Comparison of clonally expanded CD8$^+$ EM cell populations among CD8$^+$ T cells from nullipara and parous women in PBMCs **(A)** and decidua **(B)**.
